# Development of an X-ray imaging system to prevent scintillator degradation for white synchrotron radiation

**DOI:** 10.1107/S1600577518003193

**Published:** 2018-04-24

**Authors:** Tunhe Zhou, Hongchang Wang, Thomas Connolley, Steward Scott, Nick Baker, Kawal Sawhney

**Affiliations:** a Diamond Light Source Ltd, Harwell Science and Innovation Campus, Didcot OX11 0DE, UK

**Keywords:** white-beam detector, fast imaging, phase contrast, scintillators, X-ray exposure, inert gas

## Abstract

The high flux of the white X-ray beams from third-generation synchrotron light sources can significantly benefit the development of high-speed X-ray imaging, but can also bring technical challenges to existing X-ray imaging systems. One prevalent problem is that the image quality deteriorates because of dust particles accumulating on the scintillator screen during exposure to intense X-ray radiation. Here, this problem has been solved by embedding the scintillator in a flowing inert-gas environment. It is also shown that the detector maintains the quality of the captured images even after days of X-ray exposure. This modification is cost-efficient and easy to implement. Representative examples of applications using the X-ray imaging system are also provided, including fast tomography and multimodal phase-contrast imaging for biomedical and geological samples.

## Abstract 2

An X-ray imaging system has been designed to overcome the challenges from the high flux of white-beam radiation, including degradation of image quality caused by accumulated contamination on the scintillator during intense X-ray exposure.

## 1. Introduction   

Third-generation synchrotron sources generate hard X-rays with significantly higher flux than laboratory X-ray sources. Nevertheless, with monochromatic X-rays selected by crystal monochromators, only a small portion of the flux can be used, and the lowered flux inevitably limits the data acquisition speed. For many X-ray imaging applications, the requirement for temporal coherence is moderate, in which case the flux can be significantly increased by using multilayer monochromators with a larger spectral bandpass. However, the image quality often suffers from the irregular stripe modulations caused by imperfections in the multilayers (Rack *et al.*, 2010 ▸), and the stripes are difficult to remove by flat-field correction in post-processing. To maximize the usage of the high flux of the synchrotron radiation and further decrease the data acquisition time, a pink (with filters) or white beam can be used by removing the monochromator and other X-ray optics. In addition, image artefacts caused by imperfections in the beamline optics can also be avoided by using a pink or white beam. Taking advantage of such a high-flux beam, time-resolved X-ray imaging opened many new avenues of scientific endeavor. Examples include studies of fluid dynamics by X-ray imaging, whereby the inaccuracy from the refraction and reflection in visible light photography can be avoided (Lee *et al.*, 2013 ▸); imaging the dynamic behavior under extreme conditions of materials that are nontransparent in visible light (Eakins & Chapman, 2014 ▸; Luo *et al.*, 2012 ▸; Workman *et al.*, 2010 ▸); and four-dimensional imaging with high-speed X-ray tomography revealing the dynamics of the internal three-dimensional structure of the samples (Walker *et al.*, 2014 ▸; Baker *et al.*, 2012 ▸).

To further enhance the image contrast and retrieve complementary information to absorption-contrast imaging, diverse X-ray phase-contrast imaging techniques have been developed over the last two decades. Importantly, several of the phase-contrast imaging techniques, such as the propagation-based, the grating-based and the speckle-based methods, are compatible with a polychromatic beam and can therefore benefit from the high flux of white beam radiation (Wilkins *et al.*, 1996 ▸; Momose *et al.*, 2009 ▸; Wang, Kashyap, Cai *et al.*, 2016 ▸).

When using a white beam, current X-ray imaging systems face several challenges. The high flux and high energy of the beam can easily damage the scintillator, objective and other instruments in the beam path through excessive heating or radiation damage. The scintillator screen can become electrostatically charged because of the intense ionizing radiation. As a result, dust particles are attracted to the screen, degrading the image in a non-systematic manner and in some cases appearing as over-saturated spots which cannot be flat-field corrected. Scattered high-energy photons directly striking the camera sensor can also lead to saturated white spots (commonly known as ‘zingers’). An X-ray imaging system designed in order to overcome some of these problems is presented in this paper. We hope it will benefit other research groups in the community facing similar challenges. Experimental results from a tomographic scan and from a scan using the speckle-based phase-contrast technique are presented as demonstrations of potential applications of the X-ray imaging system.

## 2. Experimental setup and detector design   

**Figure 1:**
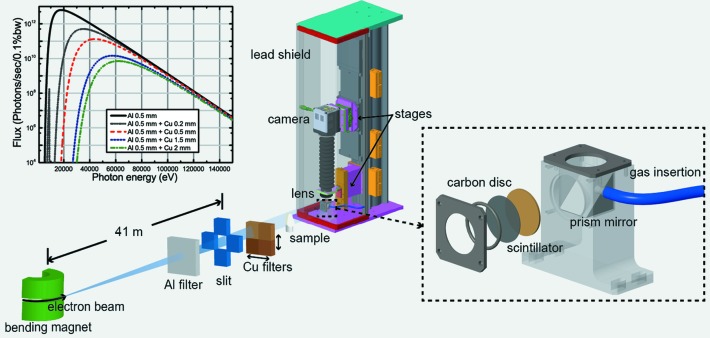
Illustration of the beamline imaging setup and camera (not to scale), and simulated spectra for a BM source at Diamond with different filters.

Tests of the detector were conducted at the bending magnet (BM) beamline B16 (Sawhney *et al.*, 2010 ▸) at Diamond Light Source (Diamond). The BM source produces a continuous spectrum of X-rays with a critical energy of 8.4 keV, with the spectrum extending to beyond 100 keV on the high-energy side. The experimental layout is illustrated in Fig. 1 ▸. Raw emission, with no narrowband spectral filtering from monochromators or focusing optics, was used. A 0.5 mm-thick aluminium attenuator was used to protect the downstream components in the beam path, such as beryllium and Kapton windows, from excessive radiation. Several beam slits were placed at various positions along the beam path to reduce the stray light and heat load on components placed upstream of the camera. Filters with different thicknesses and materials could be moved into the beam, with motorized stages prior to the sample, to tune the peak energy of the broadband beam. Spectra simulated with the *X-ray Oriented Program* (*XOP*) (Sanchez del Rio & Dejus, 2004 ▸), using aluminium and copper filters, are given as examples of this approach in Fig. 1 ▸. The simulation shows that the flux would be dramatically reduced by increasing the thickness of the copper filter, and the peak energy of the spectra would shift towards a higher energy region. Pre-hardened X-rays may be preferred, especially for samples that are thick or contain high-atomic-number materials, as the low-energy photons only increase the absorbed dose in the samples yet contribute little to achieving image contrast. The BM source was 41 m from the beam slit, and 47.5 m from the detector, between which the sample to be imaged could be placed flexibly. The camera and optics housing were lined with a lead shield protecting them from scattered X-rays and to reduce the bright pixels caused by scattered high-energy photons.

**Figure 2:**
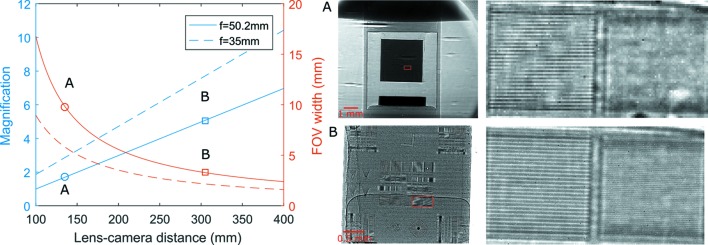
Theoretical calculations of magnifications and corresponding widths of FOV for different distances between the lens and the camera for two lenses. Images of a resolution sample taken at positions A and B are given, showing the line patterns with widths of 4 and 2 µm.

The design of the detector (Fig. 1 ▸) follows the concept of the indirect detector approach (Bonse *et al.*, 1991 ▸; Stampanoni *et al.*, 2002 ▸; Koch *et al.*, 1998 ▸), with the scintillator imaged by a CMOS camera *via* a lens. The camera and the lens are each independently mounted on a linear stage. By adjusting the positions of the lens and the camera, the geometric magnification can be varied continuously without the need to change to objectives with different magnifications. An additional goniometric stage is mounted in order to align the orientation of the camera sensor to the rotation axis of the sample stage for tomographic scans. With the current lens (50 mm *f*/2.8 Schneider macro lens) and motion-stages (150 mm and 25 mm travel range), the magnification can be varied between 1.7 and 5.1, shown as positions A and B in Fig. 2 ▸. Two radiographic images of a resolution sample (Micro-CT Bar Pattern NANO) at these two different geometrical magnifications are also given in Fig. 2 ▸. The patterns with linewidths of 4 and 2 µm are shown in the images, where we can see that the finer line pattern with a linewidth of 2 µm is at the limit of the resolution with the higher magnification setting. If a higher magnification is desired with the same overall camera dimensions, then a macro lens with shorter focal length can be used, as shown by the dashed lines in the plot in Fig. 2 ▸. Compared with microscope objectives with relatively small magnifications, a macro lens has a larger numerical aperture, and hence higher collection efficiency. Long-working-distance microscope objectives with higher magnifications are good alternatives for applications that require even higher spatial resolution. The pco.edge camera has a pixel size of 6.5 µm and 2560 × 2160 pixels. The field of view (FOV) and effective pixel size changes along with the varying of magnification. This tradeoff between a larger FOV and higher spatial resolution is illustrated in the plot in Fig. 2 ▸. The maximum frame rate of the camera is 100 frames s^−1^ at full resolution. With a reduction of the total pixel number by using only a region of the sensor, the frame rate can be increased. Depending on the application, existing cameras on the market can reach frame rates as high as MHz and capture even single-bunch X-ray images (Etoh *et al.*, 2003 ▸; Olbinado *et al.*, 2017 ▸), with a sacrifice of either FOV or spatial resolution.

The scintillator used was LuAG:Ce from Crytur with a thickness of 80 µm and a diameter of 25 mm. A glassy carbon window was mounted in front of the scintillator to block visible light and minimize contamination of the external surface by dust. Olbinado *et al.* have shown that LuAG:Ce is one of the scintillator materials whose emission spectra is best matched to CMOS sensor quantum efficiency (Olbinado *et al.*, 2017 ▸). The thickness of the scintillator affects the spatial resolution and the conversion efficiency, which can be altered depending on the requirement of the experimental application. It should be noted that the scintillator can be replaced with others that have shorter decay times, such as LYSO:Ce or LuI3:Ce (Marton *et al.*, 2014 ▸; Pidol *et al.*, 2004 ▸), which are more suitable for ultrafast imaging.

**Figure 3:**
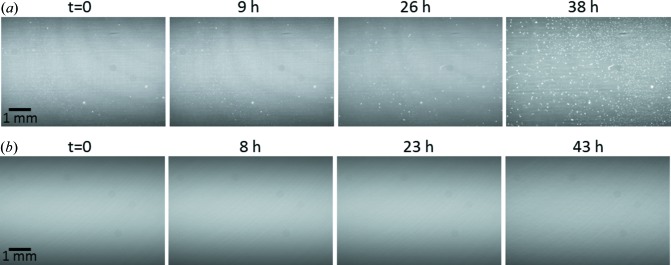
Flat-field image taken after scintillator is exposed with a pink beam (*a*) without and (*b*) with nitro­gen gas flow.

The scintillator was mounted on a 45° prism mirror cage, so that the lens and the camera could be mounted transverse to the X-ray beam direction allowing it to be protected from radiation damage caused by the X-rays that passed through the scintillator. Dry nitro­gen gas with a positive gas pressure constantly flowed into the mirror cage with a slow speed so that the dust from the surrounding environment could not enter the mirror cage and accumulate on the charged scintillator screen during X-ray exposures. Gas flow is easier to handle and more cost-efficient than a vacuum environment for the scintillator. As can be seen from the example given in Fig. 3 ▸(*a*), without any gas flow, a significant amount of dust accumulated after using the camera with a pink beam for 38 h, which manifested as clouds of white spots in the X-ray image. The image quality was significantly degraded compared with that at the beginning of the test. In this case, the scintillator normally needed to be cleaned, or sometimes replaced. In Fig. 3 ▸(*b*), when the gas flow was on, the FOV was still clean after being used for 43 h. This gave a much more stable image background for long duration or high-throughput experiments, thereby enabling a better comparison against time for applications that involve dynamically changing samples. The different efficiencies of individual scintillators and the slight variation in camera focus when manually switching the scintillators can also bring inconsistency into the collected images, which is best avoided for most applications. Eliminating the need to change and clean scintillators during experiments also offers benefits of reduced costs and downtime.

## 3. Applications   

### 3.1. Fast imaging   

With the improved X-ray imaging system installed, we will be able to take full advantage of the much higher total flux of a white or pink beam compared with a monochromatic beam, and the required acquisition time can be significantly reduced. A more efficient use of beam time is therefore possible, as well as lessening the risk of artefacts from instabilities in fixation of sample or mechanical drifts during long exposures. This is especially valuable for experiments including tomographic scans and multi-dimensional raster scans. The high flux of the white beam and a camera that is compatible with such high radiation also makes it possible for fly scans (continuous rotation mode) (Kalender *et al.*, 1990 ▸; Lak *et al.*, 2008 ▸), or time-resolved experiments.

**Figure 4:**
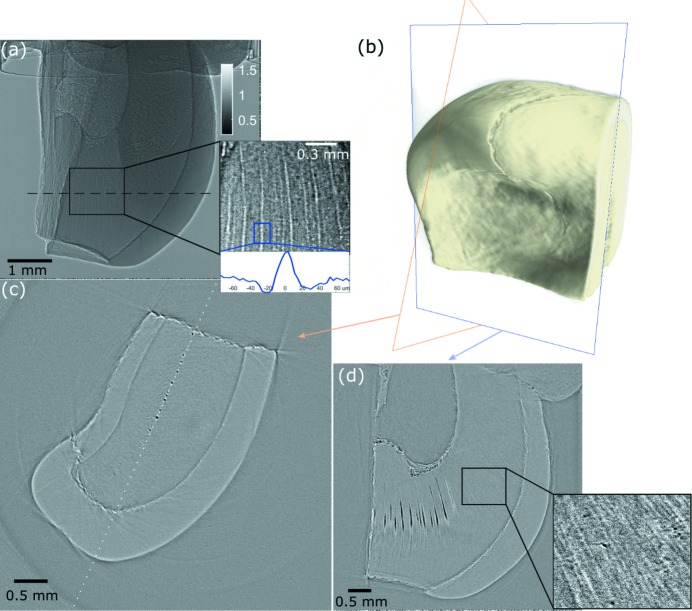
(*a*) Projection image of half of a primary tooth with an exposure time of 50 ms. The inset shows the dentine of the tooth with microcanals. (*b*) Three-dimensional rendering of the tomography. (*c*) Reconstructed transverse slice taken from the position indicated by the dashed line in (*a*), revealing the microcanals aligned in the mesiodistal direction. (*d*) Vertical slice taken from the dotted line in (*c*) along the microcanals, with inset showing the tubules in the dentine.

A tomographic scan was conducted at B16 during camera tests, and is shown in Fig. 4 ▸. Half of a human primary tooth was imaged with an exposure time of 50 ms for each projection with 601 projections taken over 180°. The effective pixel size was 3.8 µm. The beam was filtered by 0.5 mm Al and 0.5 mm Cu, leading to a peak X-ray energy of 43 keV as shown in Fig. 1 ▸. Fig. 4 ▸(*a*) shows a projection image from the scan. The image is flat-field and dark image corrected. The standard deviation of the background is 0.025. The inset shows part of the dentine in the tooth, where giant tubules or microcanals can be observed (Agematsu *et al.*, 1990 ▸, 2005 ▸; Sumikawa *et al.*, 1999 ▸). The enamel, dentine and pulp of the tooth are clearly distinguishable, thanks to edge-enhancements from phase contrast. Fig. 4 ▸(*b*) shows the three-dimensional rendering of the tomographic scan. Fig. 4 ▸(*c*) shows a transverse slice at the position indicated by the dashed line in Fig. 4 ▸(*a*), showing that the microcanals are aligned along the mesiodistal direction, in agreement with previous studies (Sumikawa *et al.*, 1999 ▸; Agematsu *et al.*, 2005 ▸). Fig. 4 ▸(*d*) is a vertical slice taken from the dotted line in Fig. 4 ▸(*c*), showing the pulp chamber and the propagating direction of the microcanals. From the inset with enhanced contrast, we can also see the structure of tubules in the dentine.

Previous studies on the microstructure of teeth have been mostly carried out using light microscopy or scanning electron microscopy (SEM). Both methods require the sample to be cut into thin slices, in order to observe the surface structure. Prior knowledge is required to find the region of interest for sample preparation. Drying and gold sputtering are also normally needed for SEM observations (Janda, 1995 ▸). X-rays, on the other hand, can image the internal three-dimensional structure non-destructively. The sample can be preserved in a more natural state to avoid artefacts and damage that can be introduced by the sample preparation process; therefore, more realistic information about the sample can be extracted. Fast and large-FOV tomography can also be applied as a pre-scan before the destructive sample preparation process for other imaging tools with higher resolution, such as SEM, or more recently, ptychography (Zanette, Enders *et al.*, 2015 ▸), so that sample cutting can be controlled and targeted at the region of interest.

### 3.2. High-energy phase-contrast imaging   

**Figure 5:**
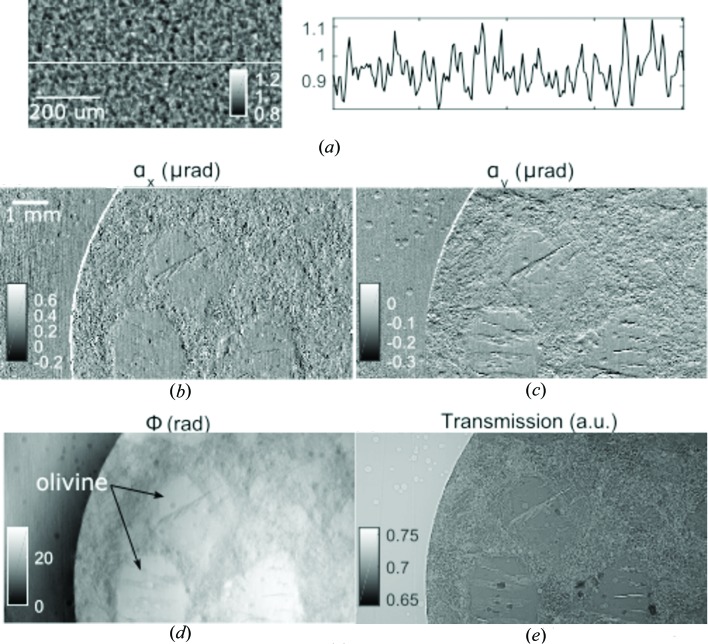
(*a*) Flat-field-corrected speckle image generated by the sandpaper and the profile taken along the line. (*b*) Refraction angles in horizontal and (*c*) in vertical directions, (*d*) integrated phase, and (*e*) transmission image of a volcanic rock sample.

When the white-beam from the BM is filtered, low-energy photons are absorbed and a spectrum with a higher average energy is achieved. Materials that have high atomic numbers can be imaged and characterized, for example, with phase-contrast imaging methods (Spanne *et al.*, 1999 ▸; Wang, Kashyap, Cai *et al.*, 2016 ▸; Diemoz *et al.*, 2016 ▸; Willner *et al.*, 2013 ▸). With multimodal phase-contrast imaging, results about phase, absorption and scattering can be obtained simultaneously, which offers complementary information to the material properties compared with commonly used attenuation-contrast imaging (Pfeiffer *et al.*, 2008 ▸; Berujon, Wang *et al.*, 2012 ▸; Modregger *et al.*, 2016 ▸; Pagot *et al.*, 2003 ▸). In the example given here, the speckle-based phase-contrast technique with one-dimensional scan mode has been applied (Wang *et al.*, 2015 ▸, 2016 ▸), where a stack of sandpaper (P800) is used as the static diffuser. An image of the sandpaper is given in Fig. 5 ▸(*a*), with the random structures resembling ‘speckles’. The effective pixel size was 3.8 µm. In this experiment, 0.5 mm-thick Al and 1.5 mm-thick Cu filters were used, which gave a hardened spectrum with peak energy at 57.5 keV, as shown in Fig. 1 ▸. In this case, the visibility of the speckle pattern, defined as the normalized standard deviation here (Zanette, Zdora *et al.*, 2015 ▸), is about 7%. The speckle size calculated as the FWHM of the autocorrelation is 12 µm. Other materials, such as steel wool (Wang, Kashyap, Cai *et al.*, 2016 ▸), can also be used as diffuser, which can give a different visibility and speckle size.

The principle of the speckle-based phase-contrast imaging is to measure the refraction of the X-ray beam induced by the sample, by tracking the shift of the speckle pattern. With the proportional relation 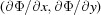 = 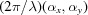, where 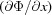 and 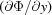 are the phase gradients in the horizontal and vertical, respectively, 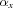 and 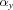 are the refraction angles and λ is the wavelength of the X-rays, the phase shift Φ can be calculated by two-dimensional integration (Morgan *et al.*, 2012 ▸; Berujon, Ziegler *et al.*, 2012 ▸). Simultaneously, transmission and dark-field images can also be obtained, from the changes in the intensity and the visibility of the speckle pattern induced by the sample, revealing information about absorption and scattering (Berujon, Wang *et al.*, 2012 ▸).

As a demonstration of the potential applications in geological science, we selected one picrite (an important chemical end-member magma) sample from Iceland. It is composed of olivine macrocrystals set in a microcrystalline matrix that also contains some olivine. The thickness of the sample is 530 µm. Three-dimensional textural information is key to understanding cooling histories, and also to contextualize the chemical information of the materials. Attenuation contrast works well for distinguishing between crystals and air for porous samples. However, because of the chemical similarity between the macrocrystal and the matrix there is very little difference in attenuation, which means that phase contrast is highly valuable (Polacci *et al.*, 2010 ▸). The development of this imaging system allows for new dynamic experiments to be designed that test long-held assumptions regarding the origin and evolution of these and other magmas. As shown in Fig. 5 ▸, the multimodal images of the picrite sample are retrieved using the speckle-based technique. The transmission image is shown in Fig. 5 ▸(*e*), and it exhibits poor absorption contrast between the olivine and the host groundmass. While this image can be interpreted by eye, it is difficult to segment digitally. The phase contrast image in Fig. 5 ▸(*d*), integrated from the horizontal and vertical refraction angles in Figs. 5 ▸(*b*) and 5(*c*), produces better contrast which can be segmented more easily. Further work is under way to apply this technique to tomographic imaging, where virtual slices can be reconstructed without the need for preparing thin sections of the sample. Combined with tomography, multimodal images will allow quantitative retrieval of the complex refractive indices of the materials in the sample, and can be applied for material characterization and quantitative analysis (Zanette, Zdora *et al.*, 2015 ▸). Multimodal imaging also offers the extra scattering information that traditional attenuation imaging cannot. For example, it can be used to study water transportation across construction materials (Yang *et al.*, 2015 ▸). With the development of the improved white-beam detecting system, such scientific applications are also possible at beamline B16. By enhancing the applicability and utility of BM beamlines for imaging, synchrotron X-ray users have expanded the opportunity to conduct a greater number of experiments, by providing an alternative to heavily over-subscribed insertion-device-based imaging beamlines.

## 4. Conclusion   

A white-beam imaging system has been designed, tested and established at the B16 beamline at Diamond. Continuous nitro­gen flow in the space between the scintillator and the reflecting mirror has proved to be an effective way of preventing accumulation of dust particles on the scintillator, which would otherwise significantly degrade image quality over time. Similar concepts can be applied to other existing X-ray imaging systems. The spatial resolution and FOV can be adjusted continuously by varying the positions of the lens and the camera with motorized stages, without the need to change to objectives with different magnifications. Demonstrations for fast tomography and multimodal phase-contrast imaging show that the X-ray imaging system developed will find wide applications, such as in the biomedical and geological sciences. With the further development of the detector, the white-beam imaging system will be routinely used, for example to study dynamic behavior of samples in fly-scan mode. The design of the imaging system is cost-efficient and flexible, and can be implemented easily on other synchrotron beamlines that provide intense X-ray flux.

## References

[bb1] Agematsu, H., Abe, S., Shiozaki, K., Usami, A., Ogata, S., Suzuki, K., Soejima, M., Ohnishi, M., Nonami, K. & Ide, Y. (2005). *Bull. Tokyo Dent. Collect.* **46**, 7–15. 10.2209/tdcpublication.46.7 16285599

[bb2] Agematsu, H., Watanabe, H., Yamamoto, H., Fukayama, M., Kanazawa, T. & Miake, K. (1990). *Bull. Tokyo Dent. Collect.* **31**, 163–173. 2131169

[bb3] Baker, D. R., Brun, F., O’Shaughnessy, C., Mancini, L., Fife, J. L. & Rivers, M. (2012). *Nat. Commun.* **3**, 1135. 10.1038/ncomms2134 23072805

[bb4] Berujon, S., Wang, H. & Sawhney, K. (2012). *Phys. Rev. A*, **86**, 063813.

[bb5] Bérujon, S., Ziegler, E., Cerbino, R. & Peverini, L. (2012). *Phys. Rev. Lett.* **108**, 158102. 10.1103/PhysRevLett.108.158102 22587288

[bb6] Bonse, U., Nusshardt, R., Busch, F., Pahl, R., Kinney, J. H., Johnson, Q. C., Saroyan, R. A. & Nichols, M. C. (1991). *J. Mater. Sci.* **26**, 4076–4085.

[bb7] Diemoz, P. C., Bravin, A., Sztrókay-Gaul, A., Ruat, M., Grandl, S., Mayr, D., Auweter, S., Mittone, A., Brun, E., Ponchut, C., Reiser, M. F., Coan, P. & Olivo, A. (2016). *Phys. Med. Biol.* **61**, 8750–8761. 10.1088/1361-6560/61/24/8750 27893445

[bb8] Eakins, D. E. & Chapman, D. J. (2014). *Rev. Sci. Instrum.* **85**, 123708. 10.1063/1.4904275 25554302

[bb9] Etoh, T. G., Poggemann, D., Kreider, G., Mutoh, H., Theuwissen, A. J. P., Ruckelshausen, A., Kondo, Y., Maruno, H., Takubo, K., Soya, H., Takehara, K., Okinaka, T. & Takano, Y. (2003). *IEEE Trans. Electron Devices*, **50**, 144–151.

[bb10] Janda, R. (1995). *Biomaterials*, **16**, 209–217. 10.1016/0142-9612(95)92119-q 7748997

[bb11] Kalender, W. A., Seissler, W., Klotz, E. & Vock, P. (1990). *Radiology*, **176**, 181–183. 10.1148/radiology.176.1.2353088 2353088

[bb12] Koch, A., Raven, C., Spanne, P. & Snigirev, A. (1998). *J. Opt. Soc. Am. A*, **15**, 1940–1951.

[bb13] Lak, M., Néraudeau, D., Nel, A., Cloetens, P., Perrichot, V. & Tafforeau, P. (2008). *Microsc. Microanal.* **14**, 251–259. 10.1017/S1431927608080264 18312722

[bb14] Lee, J. S., Weon, B. M. & Je, J. H. (2013). *J. Phys. D*, **46**, 494006.

[bb15] Luo, S. N., Jensen, B. J., Hooks, D. E., Fezzaa, K., Ramos, K. J., Yeager, J. D., Kwiatkowski, K. & Shimada, T. (2012). *Rev. Sci. Instrum.* **83**, 073903. 10.1063/1.4733704 22852700

[bb16] Marton, Z., Nagarkar, V. V., Miller, S. R., Brecher, C., Bhandari, H. B., Kenesei, P., Ross, S. K., Almer, J. D. & Singh, B. (2014). *J. Phys. Conf. Ser.* **493**, 012017.

[bb17] Modregger, P., Cremona, T. P., Benarafa, C., Schittny, J. C., Olivo, A. & Endrizzi, M. (2016). *Sci. Rep.* **6**, 30940. 10.1038/srep30940 PMC4974648 27491917

[bb18] Momose, A., Yashiro, W., Maikusa, H. & Takeda, Y. (2009). *Opt. Express*, **17**, 12540–12545. 10.1364/oe.17.012540 19654656

[bb19] Morgan, K. S., Paganin, D. M. & Siu, K. K. W. (2012). *Appl. Phys. Lett.* **100**, 124102–124104.

[bb20] Olbinado, M. P., Just, X., Gelet, J.-L., Lhuissier, P., Scheel, M., Vagovic, P., Sato, T., Graceffa, R., Schulz, J., Mancuso, A., Morse, J. & Rack, A. (2017). *Opt. Express*, **25**, 13857–13871. 10.1364/OE.25.013857 28788829

[bb21] Pagot, E., Cloetens, P., Fiedler, S., Bravin, A., Coan, P., Baruchel, J., Härtwig, J. & Thomlinson, W. (2003). *Appl. Phys. Lett.* **82**, 3421–3423.

[bb22] Pfeiffer, F., Bech, M., Bunk, O., Kraft, P., Eikenberry, E. F., Brönnimann, C., Grünzweig, C. & David, C. (2008). *Nat. Mater.* **7**, 134–137. 10.1038/nmat2096 18204454

[bb23] Pidol, L., Kahn-Harari, A., Viana, B., Virey, E., Ferrand, B., Dorenbos, P., de Haas, J. T. M. & van Eijk, C. W. E. (2004). *IEEE Trans. Nucl. Sci.* **51**, 1084–1087.

[bb24] Polacci, M., Mancini, L. & Baker, D. R. (2010). *J. Synchrotron Rad.* **17**, 215–221. 10.1107/S0909049509048225 20157274

[bb25] Rack, A., Weitkamp, T., Riotte, M., Grigoriev, D., Rack, T., Helfen, L., Baumbach, T., Dietsch, R., Holz, T., Krämer, M., Siewert, F., Meduňa, M., Cloetens, P. & Ziegler, E. (2010). *J. Synchrotron Rad.* **17**, 496–510. 10.1107/S0909049510011623 20567082

[bb26] Sanchez del Rio, M. & Dejus, R. J. (2004). *Proc. SPIE*, **5536**, 171–174.

[bb27] Sawhney, K. J. S., Dolbnya, I. P., Tiwari, M. K., Alianelli, L., Scott, S. M., Preece, G. M., Pedersen, U. K., Walton, R. D., Garrett, R., Gentle, I., Nugent, K. & Wilkins, S. (2010). *AIP Conf. Proc.* **1234**, 387–390.

[bb28] Spanne, P., Raven, C., Snigireva, I. & Snigirev, A. (1999). *Phys. Med. Biol.* **44**, 741–749. 10.1088/0031-9155/44/3/016 10211807

[bb29] Stampanoni, M., Borchert, G., Wyss, P., Abela, R., Patterson, B., Hunt, S., Vermeulen, D. & Rüegsegger, P. (2002). *Nucl. Instrum. Methods Phys. Res. A*, **491**, 291–301.

[bb30] Sumikawa, D. A., Marshall, G. W., Gee, L. & Marshall, S. J. (1999). *Pediatr. Dent.* **21**, 439–444. 10633518

[bb31] Walker, S. M., Schwyn, D. A., Mokso, R., Wicklein, M., Müller, T., Doube, M., Stampanoni, M., Krapp, H. G. & Taylor, G. K. (2014). *PLoS Biol.* **12**, e1001823. 10.1371/journal.pbio.1001823 PMC3965381 24667677

[bb32] Wang, H., Kashyap, Y., Cai, B. & Sawhney, K. (2016). *Sci. Rep.* **6**, 30581. 10.1038/srep30581 PMC4964655 27466217

[bb33] Wang, H., Kashyap, Y. & Sawhney, K. (2015). *Opt. Express*, **23**, 23310–23317. 10.1364/OE.23.023310 26368432

[bb34] Wang, H., Kashyap, Y. & Sawhney, K. (2016). *Sci. Rep.* **6**, 20476. 10.1038/srep20476 PMC4742822 26847921

[bb35] Wilkins, S. W., Gureyev, T. E., Gao, D., Pogany, A. & Stevenson, A. W. (1996). *Nature (London)*, **384**, 335–338.

[bb36] Willner, M., Bech, M., Herzen, J., Zanette, I., Hahn, D., Kenntner, J., Mohr, J., Rack, A., Weitkamp, T. & Pfeiffer, F. (2013). *Opt. Express*, **21**, 4155–4166. 10.1364/OE.21.004155 23481949

[bb37] Workman, J., Cobble, J., Flippo, K., Gautier, D. C., Montgomery, D. S. & Offermann, D. T. (2010). *Rev. Sci. Instrum.* **81**, 10E520. 10.1063/1.3485109 21034048

[bb38] Yang, F., Griffa, M., Bonnin, A., Mokso, R. C. D. I. B., DI Bella, C., Münch, B., Kaufmann, R. & Lura, P. (2015). *J. Microsc.* **261**, 88–104. 10.1111/jmi.12319 26469285

[bb39] Zanette, I., Enders, B., Dierolf, M., Thibault, P., Gradl, R., Diaz, A., Guizar-Sicairos, M., Menzel, A., Pfeiffer, F. & Zaslansky, P. (2015). *Sci. Rep.* **5**, 9210. 10.1038/srep09210 PMC4366856 25790969

[bb40] Zanette, I., Zdora, M.-C., Zhou, T., Burvall, A., Larsson, D. H., Thibault, P., Hertz, H. M. & Pfeiffer, F. (2015). *Proc. Natl Acad. Sci. USA*, **112**, 12569–12573. 10.1073/pnas.1502828112 PMC4611602 26424447

